# The British Journals: Pathology, Practical Medicine, and Therapeutics

**Published:** 1839-10

**Authors:** 


*Edin. Med. and Surg. Journal. July,* 1839.

### PATHOLOGY, PRACTICAL MEDICINE, AND THERAPEUTICS.

*Contributions to Intra-Uterine Pathology.* Part II. *On the Inflammatory Origin of some varieties of Hernia and Malformation in the Fœtus.* By JAMES Y. SIMPSON, M.D., Fellow of the Royal College of Physicians, Edinburgh, Lecturer on Midwifery, &c.

THIS is a most important article; and we regret that we are unable to give any portion of it in our pages. It is a continuation of a former paper noticed in our Thirteenth Number (p. 284); and its object is to trace "the *indirect* morbid effects which occasionally result from peritonitis in the fœtus," described in the former part. The author here considers, 1st, the pathological relations of fœtal peritonitis in the production of certain congenital forms of umbilical, diaphragmatic, and inguinal hernia; 2dly, endeavours to trace the primary origin of some varieties of malformation among the abdominal and pelvic viscera to the same proximate cause; 3d, notices the dependance of other species of malformation upon the effects of inflammatory action in the early embryo.

We recommend this paper to the particular attention of our readers.

*Edin. Med. and Surg. Journal. July*, 1839.

---

*The Result of Experience in Sea-Scurvy, with Remarks on its Prevention and Treatment.* By ANDREW HENDERSON, M.D. Surgeon, Royal Navy.

THIS paper contains some new matter, and a good deal of information that will be valuable to his junior brother officers in the navy, and to surgeons in ships of commerce. Dr. Henderson has performed seven voyages in charge of male convicts to Van Diemen's Land and New South Wales, with results as to health which testify strongly to the excellence of his arrangements. The author, like all well-informed surgeons and captains of the present day, trusts much more to good diet, good habits, and cleanliness, than to medicaments, for the prevention of scurvy. He denies all efficacy to lemon-juice, both as a preventive and means of cure. His remedy is *nitre*, which he gives to the amount of from two to four drachms in the course of the day, dissolved in six or eight ounces of water, in divided doses. "In this way, I have often with much satisfaction remarked a visible improvement in three days; but, in general, a much longer time is required to bring about a favorable change. I have frequently known patients very much averse to the medicine at first, saying that it caused vomiting, purging, and so forth; but never knew one attempt to refuse it after the second or third day, all coming to the hospital cheerfully at the appointed time to get their allowance, and often pushing one another aside, trying to get first in for the expected dose. The relief it affords to the sinking at the pit of the stomach, so very much referred to in *purpura*, is almost incredible. Indeed, had I not so often witnessed the remarkable change for the better in a short time, I could not credit the accounts given by the patients themselves, raised as it were from death's door to a state of ease and returning health." *Edin. Med. and Sur. Journal. July*, 1839.

---

*Observations on the Treatment of Delirium Tremens, without Opium.* By T. CAHILL, M.D.

ALTHOUGH confident, from a good deal of experience, of the infinite value of opium in many cases of delirium tremens, we have ourselves witnessed so much evil from its indiscriminate and exclusive use, that we gladly receive Dr. Cahill's criticisms on the practice. The original article contains the details of seven cases, which are intended to illustrate, and do illustrate, the proposition maintained by the author, "that opium is not beneficial in many cases; in others, that it is positively injurious; and that in all a cure can be effected without its assistance."

*Dublin Journ. of Med. Science. July*, 1839.

---

*History of a singular Convulsive Disease, affecting Five Children in one family.* By ANDREW DEWAR, Surgeon, Dunfermline.

THIS paper adds another authentic chapter to the many histories of strange anomalous convulsive diseases already on record; and strikingly illustrates, also, the important points of the propagation of such affections by *imitation*, and of their cure by the old Boerhaavian remedy, *terror*. The disease was most judiciously treated by the removal of the children from their home; and they were all cured by keeping them separate there; by threatening them with the cold affusion, searing irons, &c.; except the one originally affected, who required other means for her recovery. *Edin. Med. and Sur. Journal. July*, 1839.

*On the Treatment of Strangulated Hernia, on the plan of* Dr. O'Beirne.

Case i. By J. Sawkins, Esq. Surgeon, Towcester.

Case ii. By Fred. Ellis, Esq. of the Richmond Surgical Hospital, Dublin.

Both these cases proved successful under very unfavorable circumstances; and we entirely agree in the remark appended to the latter by Dr. O'Beirne, "that no medical man can, henceforth, be considered justified in proceeding to an operation for strangulated intestinal hernia, without having previously given a full and fair trial to the mode of treatment in question. If this principle were once acted upon, it is my conviction (says Dr. O'B.) that the operation would soon become comparatively rare, and many valuable lives be saved, which would otherwise be lost."

"Including this case (says Dr. O'B.) the practice has been employed, by myself and others, in twenty-one cases of strangulated intestinal hernia, all of which are now published. In fourteen of these the patients were quickly relieved from all suffering and danger, and, in the great majority of instances, after all other means have failed, and the operation had been decided upon. Of the seven cases in which the plan failed, the post-mortem examination in three demonstrated that they were not relievable by operation, or by any means short of divine; while, in two others, bands, adhesions, and other causes of failure were discovered during the performance of the operation; and in the two remaining there was the clearest evidence of the plan not having been fairly tried." *Lancet. July* 6*th and* 27*th*, 1839.

---

*Case of Section of the Hamstring Tendons, for the Cure of contracted Knee-joint.* By Benj. Phillips, f.r.s., Surgeon to the Marylebone Infirmary.

This is an interesting case. The contraction which originated from rheumatism was very great, of some years' standing, and had resisted all the usual means. Mr. P. divided the tendons of the biceps, semitendinosus, and (by a second operation on the third day) semimembranosus. Its result was successful.

*London Medical Gazette. July* 20, 1839.

---

*On the Treatment of Prolapsus Uteri, by means of the Actual Cautery and Caustics.*

1. *On a New and Successful Method of Treating Prolapsus Uteri.* By Benjamin Phillips, f.r.s., Surgeon to the St. Marylebone Infirmary.

2. *Letter from* Dr. Evory Kennedy, Master of the Dublin Lying-in Hospital, *to* Sir Benjamin Brodie, Bart., *on the use of Caustics in Prolapsus of the Uterus.*

Both these communications deserve the attention of surgeons. The principle of the proposed treatment consists in producing a permanent contraction (not obliteration) of the vagina, by means of the cicatrices resulting from the partial destruction of the mucous membrane by lunar caustic, the fuming nitric acid (Phillips), or the actual cautery (Kennedy). This mode of treatment seems preferable to the plan by excision. For the details we refer to the original papers which will well repay perusal.

*Gazette. June* 29, 1839. *Lancet. June* 8, 1839.

